# Effect of Bone Remodeling on Dental Implant Fatigue Limit Predicted Using 3D Finite Element Analysis

**DOI:** 10.54289/jdoe2200102

**Published:** 2022-03-09

**Authors:** Megha Satpathy, Yuanyuan Duan, Logan Betts, Matthew Priddy, Jason A. Griggs

**Affiliations:** 1 Department of Biomedical Materials Science, University of Mississippi Medical Centre, Jackson, MS, USA; 2 Department of Mechanical Engineering, Mississippi State University, Starkville, MS, USA

**Keywords:** Finite Element Analysis, Micro-Computed Tomography, Medical Implants, Bone Remodelling, Fatigue Fracture, Dentistry

## Abstract

**Background:**

To evaluate the effect of bone remodelling around a reduced-diameter dental implant on its fatigue limit using finite element analysis (FEA).

**Methods:**

A dental implant assembly, which included a reduced-diameter dental implant (Biomet-3i external hex), an abutment (GingiHue^®^) and a connector screw (Gold-Tite Square screw), was scanned using micro-computed tomography (Skyscan 1172). Its dimensions were measured using Mimics (Materialise) and an optical microscope (Keyence). The digital replicas of the physical specimens were constructed using SOLIDWORKS (Dassault Systems). A cylindrical bone specimen holder with two layers (cortical and cancellous bone) was designed in SOLIDWORKS. Two assemblies were created: (a) Model 1: Having non-remodelled bone; (b) Model 2: Cancellous bone remodelled at the regions adjacent to the implant screw threads. FEA was performed in ABAQUS (SIMULIA). In Model 1, the Young’s modulus of cortical and cancellous bone were 20 GPa and 14 GPa, respectively. For Model 2, the region of the cancellous bone adjacent to the implant screw threads was assigned a Young’s modulus of 20 GPa. fe-safe (SIMULIA) was used to estimate the fatigue limit.

**Results:**

The maximum von Mises stress under 100 N load was 439.9 MPa for both models 1 and 2 and was located at the connector screw. The fatigue limit was 116.4 N for both models 1 and 2.

**Conclusions:**

The results suggest that implant fatigue resistance tested according to ISO 14801 may be accurately predicted without bothering to simulate the non-homogeneous stiffness that occurs at the bone-implant interface in the clinical case.

## Introduction

The global market size of dental implants was valued at USD 3.77 billion in 2016, with a compound annual growth rate of 7.7% over the forecast period [1]. Dental implants find their usage in a variety of applications, such as orthodontic mini-implants, single tooth implant restoration, implant supported overdentures, and so on.

Reduced-diameter implants are necessary for replacement of teeth with small cervical diameters. However, they suffer from a high incidence of complications. A majority of reports found a strong association between narrow implant diameter and decreased clinical lifetime [1-3]. The space between teeth is not large enough for a standard diameter implant in the mandibular incisor and maxillary lateral incisor locations, hence, reduced-diameter implants are used at these locations. Therefore, one cannot substitute larger, and hence more durable, implants in place of reduced-diameter implants. Although implant failure rate is greater for smaller diameter implants, in general, an abrupt increase in failure rate has been observed at a critical threshold of about 3.75 mm diameter [4]. Our long-term goal is to better understand the fatigue failure resistance of implant designs having diameters just below this threshold. Necessary first steps were to: (1) develop a protocol for predicting the fatigue limit of an implant design using finite element modeling and (2) determine what simplifying assumptions regarding bone properties could be made without changing the predictions of the model. The purpose of this study was to achieve those first steps.

**Figure 1:**
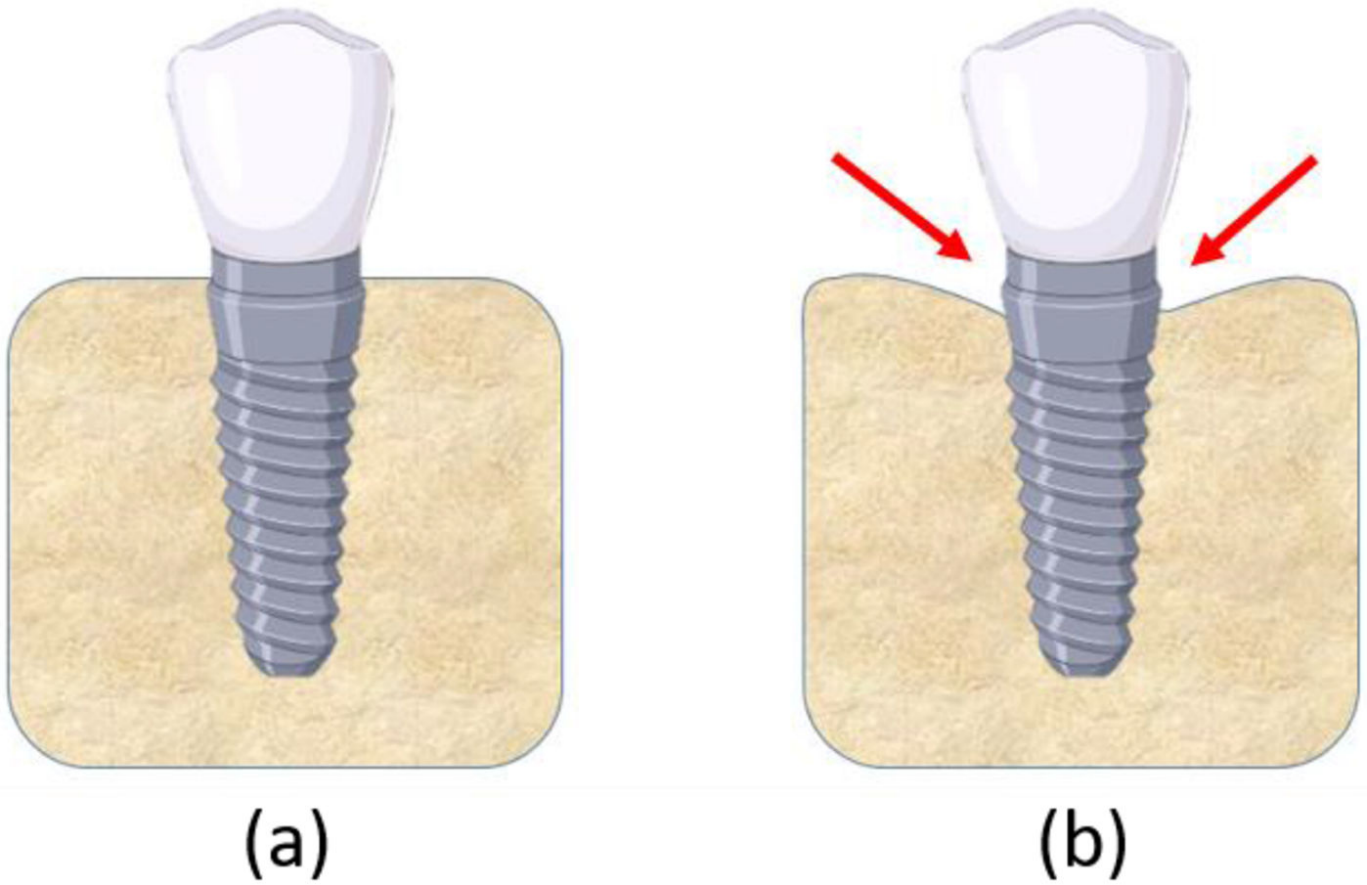
(a) Dental implant in jaw bone immediately after placement; (b) Crestal bone loss at the nominal bone level of the implant takes place over time.

Bone structure consists of two primary layers: cortical and cancellous bone. Cortical bone is referred to the hard outer layer of bone and is much denser than the cancellous bone. Cortical bone consists of multiple microscopic columns, or osteons, around the Harversian canals. Since these columns are metabolically active, bone remodelling causes changes in the orientation of the osteons and hence the stiffness of the bone in various directions. The inner layer consists of the cancellous or trabecular bone, which consists of an open cell porous network. Cancellous bone has lower stiffness than cortical bone, and is more flexible, highly vascular and contains the bone marrow where blood cells are produced [5]. Studies show that cortical and cancellous bone around a dental implant remodel in different ways. For cortical bone, crestal bone loss takes place, which refers to bone resorption around the neck of a dental implant. In general, crestal bone loss up to a depth of 1 mm could be expected within the first year of implant placement, followed by an additional 0.2 mm on an average [6]. Figure 1 shows a schematic of crestal bone loss around a dental implant collar over time.

The mode of remodelling, however, is different for cancellous bone. According to a study by Sennerby and Meredith, histological analysis of a dental implant placed in bone showed that the region of the cancellous bone adjacent to the implant screw threads transforms into a cortical bone structure over time [7]. This results in an increased stiffness of the implant-bone interface. The *in vivo* data show that the depth of remodelling is approximately three times the screw thread height. The study suggests that this healing process results in the formation of bone that leads to reinforcement of the implant-bone interface by forming bony ridges between the surrounding bone and the implant surface.

In the past few decades, finite element analysis (FEA) has become an increasingly popular tool for modelling and analysis. FEA is a numerical method that provides a mechanism to find approximate solutions to complex structural engineering problems. It is currently used for various applications, including fields like solid mechanics, industrial machinery, automotive industry, aerospace, and defence, and so on. FEA procedures can be advantageous in several aspects, since it is cost-effective, generates analysis results within a relatively short period of time, offers calculation and visualization of a wide variety of parameters including stress and temperature, allowing the designer to rapidly analyse performance and possible modifications, and so on [8,9]. For the past several years, FEA has been extensively used for medical device design and simulation as well. Its ability to accurately simulate physiological loads and generate precise results corresponding to actual clinical scenario has made it a useful tool in the field of biomedical sciences. Among many different fields, FEA has been used to predict stresses experienced by bone after a total joint arthroplasty, to study blood flow through biological tissues, analyse heat transfer and temperature patterns in different cells and tissues, for design verification of total knee replacement implant, and so on [10-13]. With the advancement of computer-aided design (CAD) and computer-aided manufacturing (CAM) technology, FEA is set to become increasingly relevant.

FEA has also been used extensively in implant dentistry. Understanding of the fundamental theory, methodology, applications, and limitations of FEA in implant dentistry helps clinicians to interpret results of FEA studies and extrapolate them to clinical situations [14]. FEA has been used to optimize implant designs, predict failure rates of different types of implants, evaluate stresses at the bone-implant interface, investigate the durability of implant-abutment and implant-prosthesis connections, etc. [15-18]. A previous study showed that FEA can accurately predict the *in vitro* fatigue lifetime of a dental implant [19]. The transfer of load from the implant to the surrounding bone may depend on various factors, including the type of loading, magnitude, angulation and frequency of loading, geometric design of the implant, bone-implant interface, type of the prosthesis, quantity and quality of the surrounding bone, and the surface characteristics of the implant, all of which can be simulated in FEA. In the present study, we evaluated the effect of bone remodelling around a reduced-diameter dental implant using 3D finite element analysis. We hypothesized that the bone remodelling would not affect the fatigue limit (the maximum bite force corresponding to infinite lifetime).

## Materials and Methods

**Figure 2:**
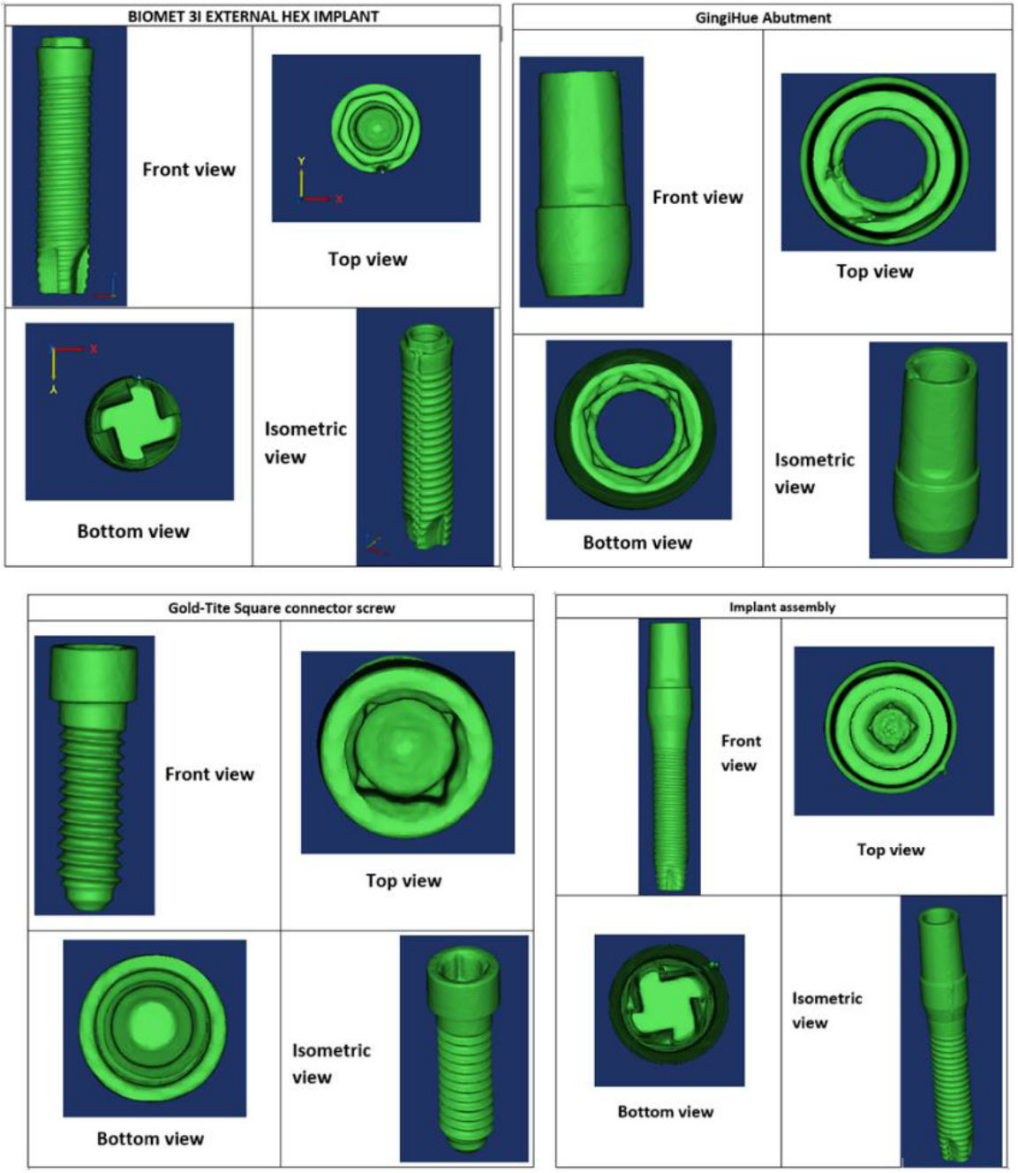
Digitial representation of the reduced-diameter implant assembly as visualized in Mimics.

The physical specimens of a dental implant assembly, which included a reduced-diameter dental implant (Biomet-3i external hex, Zimmer Biomet), an abutment (GingiHue^®^, Zimmer Biomet) and a connector screw (Gold-Tite Square screw, Zimmer Biomet), were scanned using micro-computed tomography (Skyscan 1172, Micro Photonics Inc.). The scanning parameters used were as follows: an accelerating voltage of 100 kV, current of 100 μA, exposure time of 1264 ms per frame, Al + Cu filter and rotation step at 0.7°. The x-ray beam was projected in a direction perpendicular to the long axis of the implant fixtures. The image pixel size was 34.6 μm. The x-ray projections were reconstructed to form a 3D model, which was saved as a stack of BMP-type 3D files using NRecon software (Micro Photonics Inc.). Beam hardening correction of 49% and ring artifact correction of 4 were used for the reconstruction. The 3D models were generated in Mimics (Materialise NV) through image segmentation from the stacked image data obtained from micro-CT. Mimics organizes all the imported tomograph image slices and displays objects in three cross-sectional views (axial, coronal and sagittal planes). Based on the grayscale values, the objects were modified with the help of segmentation tools. Their dimensions were then measured using a medical image processing software (Mimics, Materialise) and an optical microscope (Keyence). The length and maximum diameter of the implant were 15.12 mm and 3.40 mm, respectively. Figure 2 shows the images of the implant assembly as visualized in Mimics.

**Figure 3:**
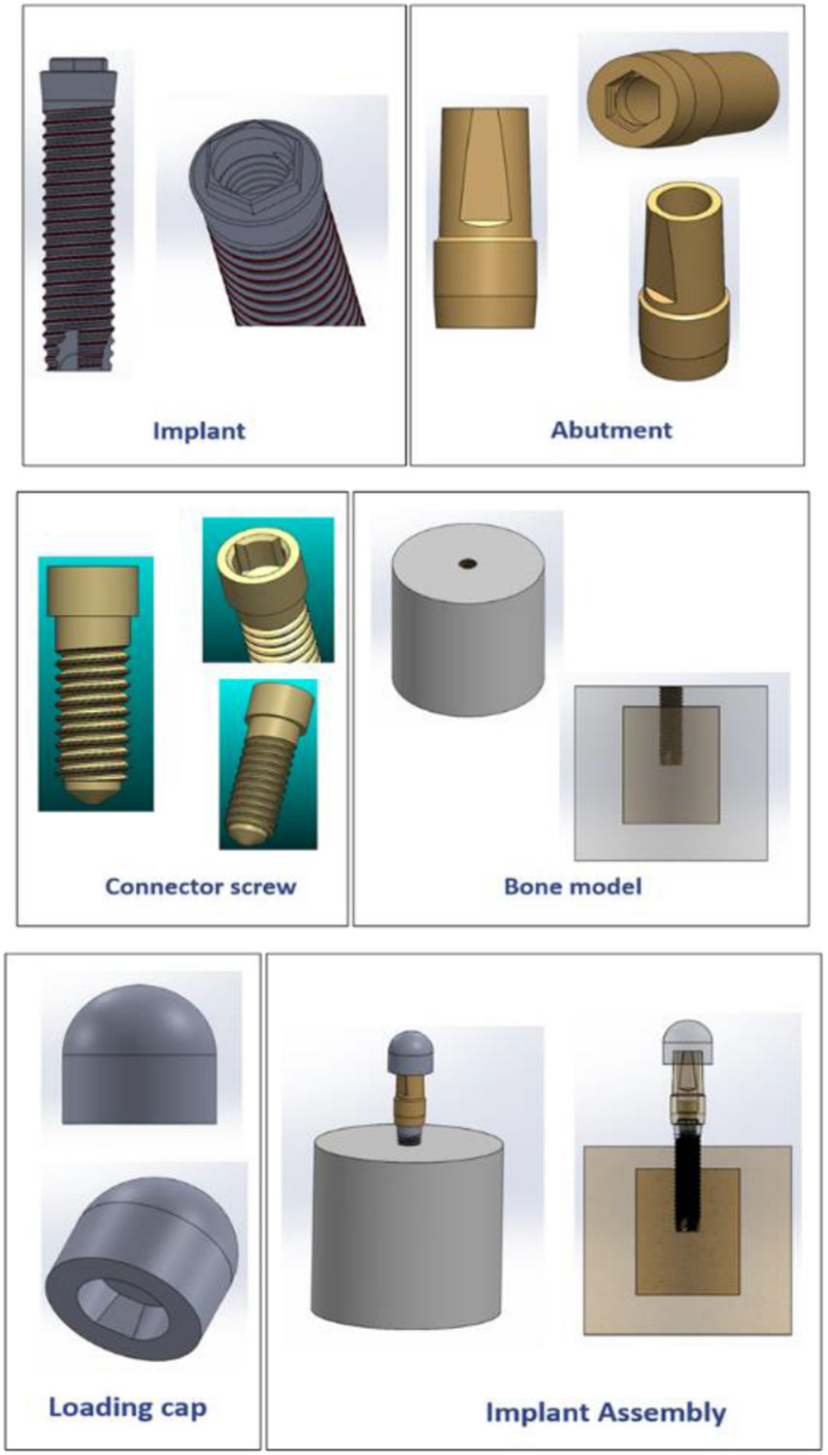
Digital replicas of the implant assembly components constructed in SOLIDWORKS.

Figure 3 shows the digital replicas of the physical specimens that were constructed using a computer-aided design software (SOLIDWORKS, Dassault Systems). A hemispherical loading cap was constructed in SOLIDWORKS to simulate a dental crown, and an 11 mm moment arm was modelled from the central point of the loading cap to the simulated bone level (as required by ISO 14801). A cylindrical bone model with two layers (cortical and cancellous bone) was also constructed in SOLIDWORKS based on the dimensions of a simulated bone specimen holder that our research group had developed in a previous study [20]. The bone crest was placed at 3 mm below the implant nominal bone level based on the requirement of ISO 14801.

Two separate models were created: Model 1 (homogeneous bone) and Model 2 (remodelled bone). In Model 1, both the cortical and cancellous bone were homogeneous, and were assigned Young’s modulus values of 20 GPa and 14 GPa, respectively [21,22]. In Model 2, the cortical bone was homogeneous, however, the cancellous bone was partitioned into two sections: (a) section 1: cancellous bone adjacent to the implant screw threads; (b) section 2: rest of the cancellous bone. Our focus was to remodel section 1 and analyse its effect on the implant fatigue limit. The study by Sennerby and Meredith (2008) indicated that the cancellous bone adjacent to the implant screw threads remodels to a structure similar to that of cortical bone with time, and the thickness of the remodeled bone was approximately three times the implant screw thread height. Since the implant screw thread height in our study was 0.15 mm, the thickness of the remodeled bone was defined as 0.45 mm.

**Figure 4:**
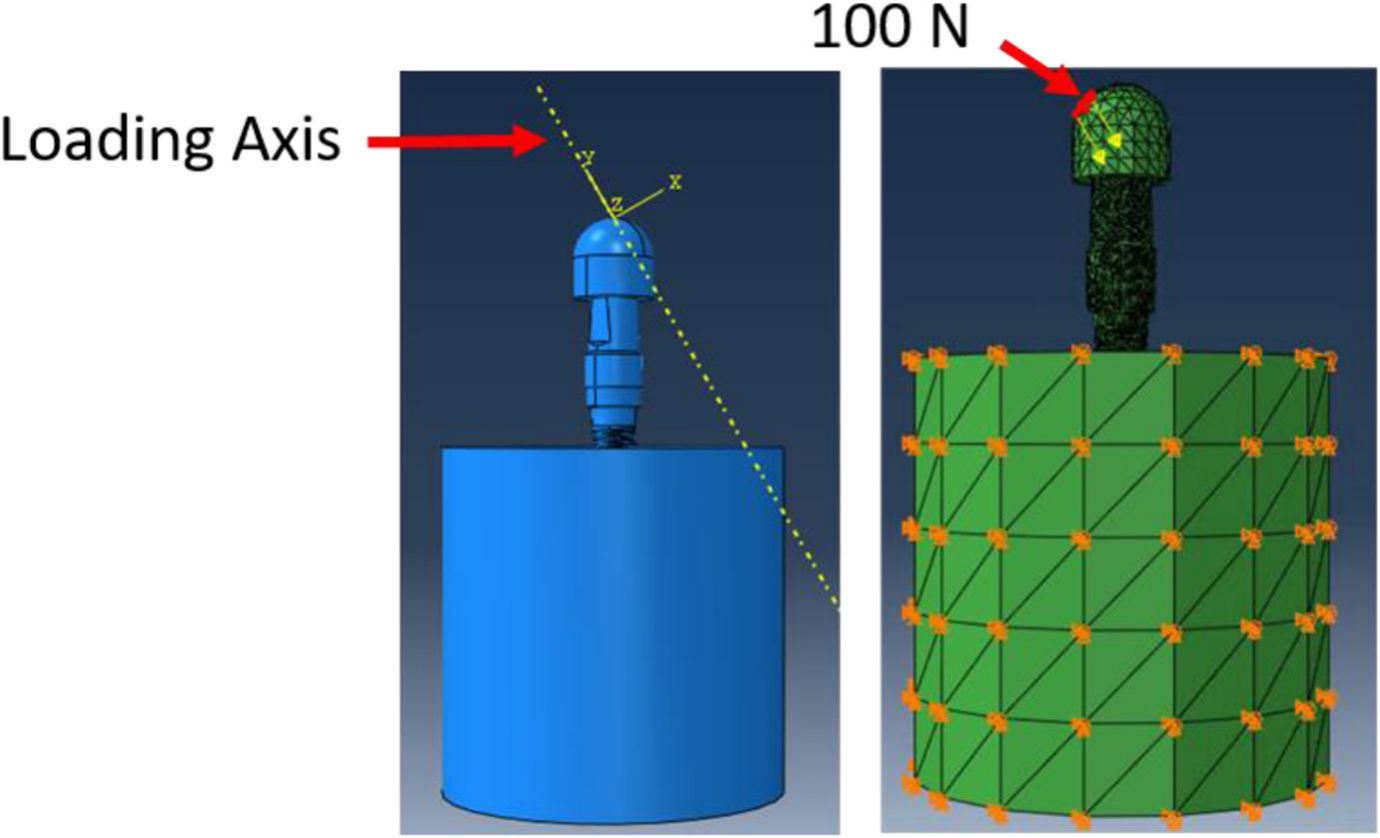
FEA was carried out with a 100 N static load applied to the loading cap at an angle of 30 degrees from the implant axis. Boundary conditions were applied on the cortical bone to constrain movement in all three directions.

Finite element analysis (FEA) was performed in ABAQUS (SIMULIA). Table 1 shows the material properties assigned to the different components in Model 1. The material properties were assumed to be homogeneous, isotropic, and linearly elastic. In Model 1, the stiffness of cortical and cancellous bone were 20 GPa and 14 GPa, respectively [21,22]. Their Poisson’s ratio was 0.3. In Model 2, section 1 of the cancellous bone was assigned a stiffness of 20 GPa and a Poisson’s ratio of 0.3, in order to simulate remodelling of this region such that its material properties are similar to that of cortical bone. Section 2, however, was comprised of the far field elements and hence, had the same material properties as the cancellous bone of Model 1. For both the models, boundary conditions were applied to the nodes on the outer surface of the cortical bone. A 100 N static load was applied to the loading cap at an angle of 30 degrees from the implant axis (ISO 14801), as shown in Figure 4. A preload of 32 N cm was applied to the connector screw, as recommended for this implant model by the manufacturer. Both models were meshed using tetrahedral element type. Convergence tests were carried out until the appropriate mesh density, which used minimum possible number of elements to achieve convergence of results, was determined for both the models. The final number of elements for each component is shown in Table 2.

Fatigue lifetime prediction was performed using fe-safe post-processing software (SIMULIA). Test loading was set as a cyclic load with a stress ratio (R) of 0.1 (ISO 14801) to simulate the physiological chewing condition. Brown-Miller criteria with Morrow mean stress correction was used for lifetime calculation [19]. The material properties were approximated using Seeger’s method with the help of the rescaling conventional monotonic ultimate tensile stress (UTS). Fatigue lifetime results were observed in an ODB file that can be displayed in the post-processor of ABAQUS.

This work was conducted from 2020 to 2022 at both the University of Mississippi Medical Center in Jackson, Mississippi and Mississippi State University in Starkville, Mississippi.

## Results

**Figure 5:**
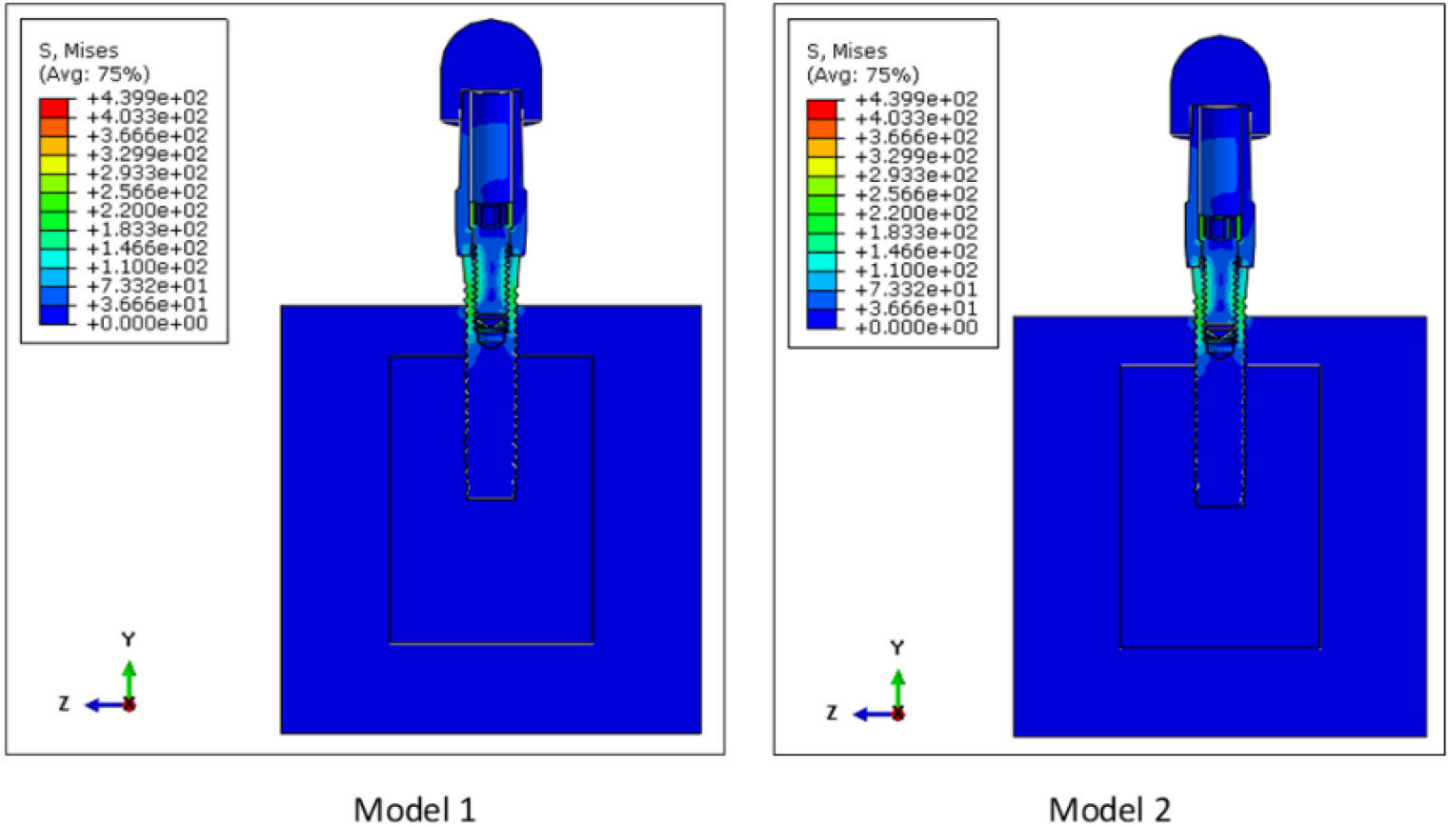
Von Mises stress distribution on Models 1 and 2. The peak stress was 439.9 MPa for both Models 1 and 2 and was concentrated at the connector screw for both models. High stresses were also observed at the implant superficial threads.

**Figure 6:**
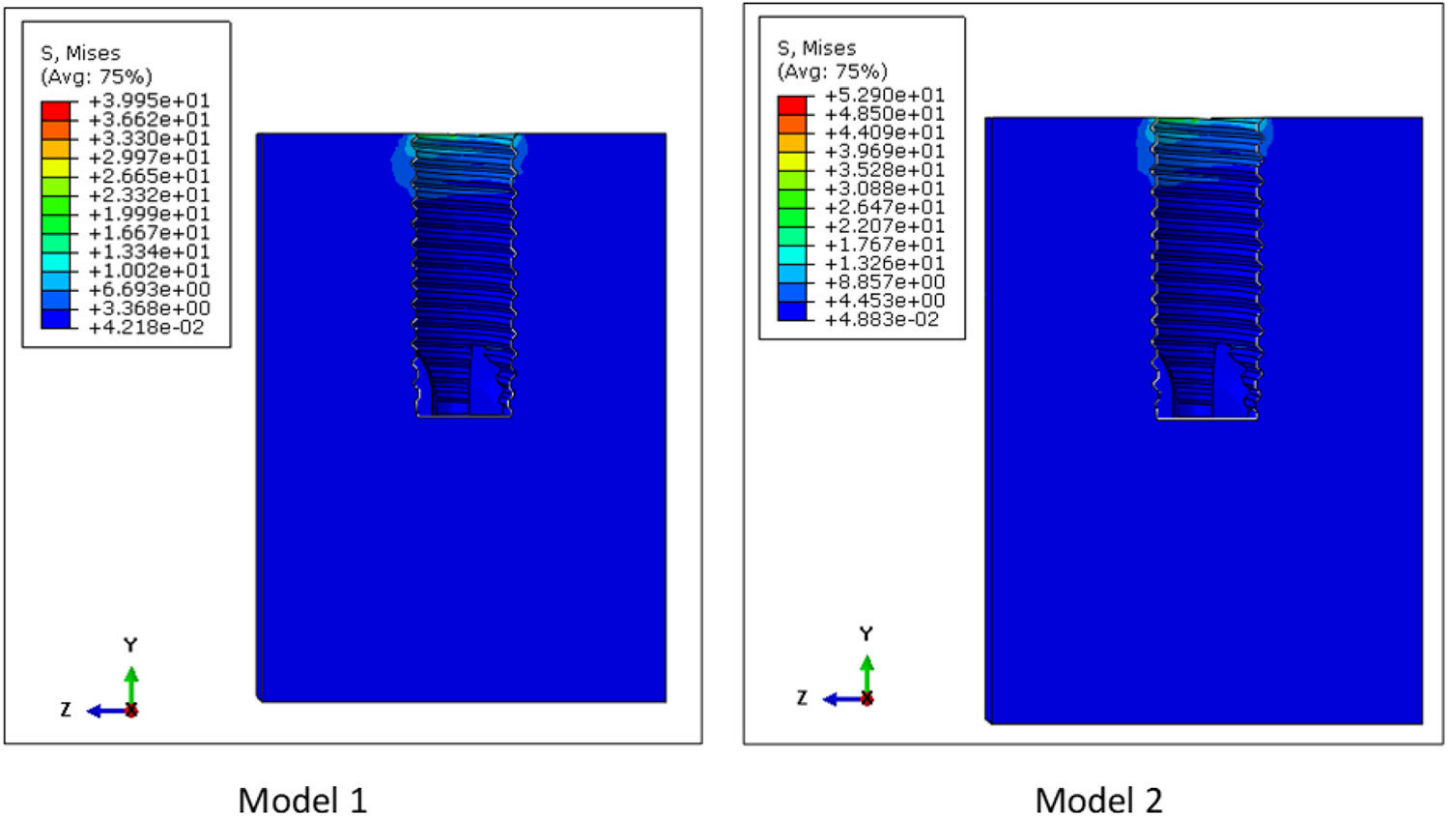
Von Mises stress distribution in the cancellous bone of Models 1 and 2. The peak stresses on the cancellous bone were 39.95 MPa and 52.90 MPa for Models 1 and 2, respectively. Higher stresses in the cancellous bone were observed at the superficial region of the inner cavity of the cancellous bone.

The von Mises stress distributions for both models are shown in Figure 5. The maximum von Mises stress was 439.9 MPa for both models 1 and 2. The location of these peak stresses were observed at the connector screw in both models. High stress concentration was also observed at the superficial threads of the implant fixture. Although the peak stresses for both these assemblies are almost the same, these two are different models. This is evidenced by looking at the von Mises stress distribution on the cancellous bone of these models, as shown in Figure 6. The peak stress value of the cancellous bone in Model 1 (39.95 MPa) is different from that of Model 2 (52.90 MPa) since the bone in Model 2 has undergone remodeling.

**Figure 7:**
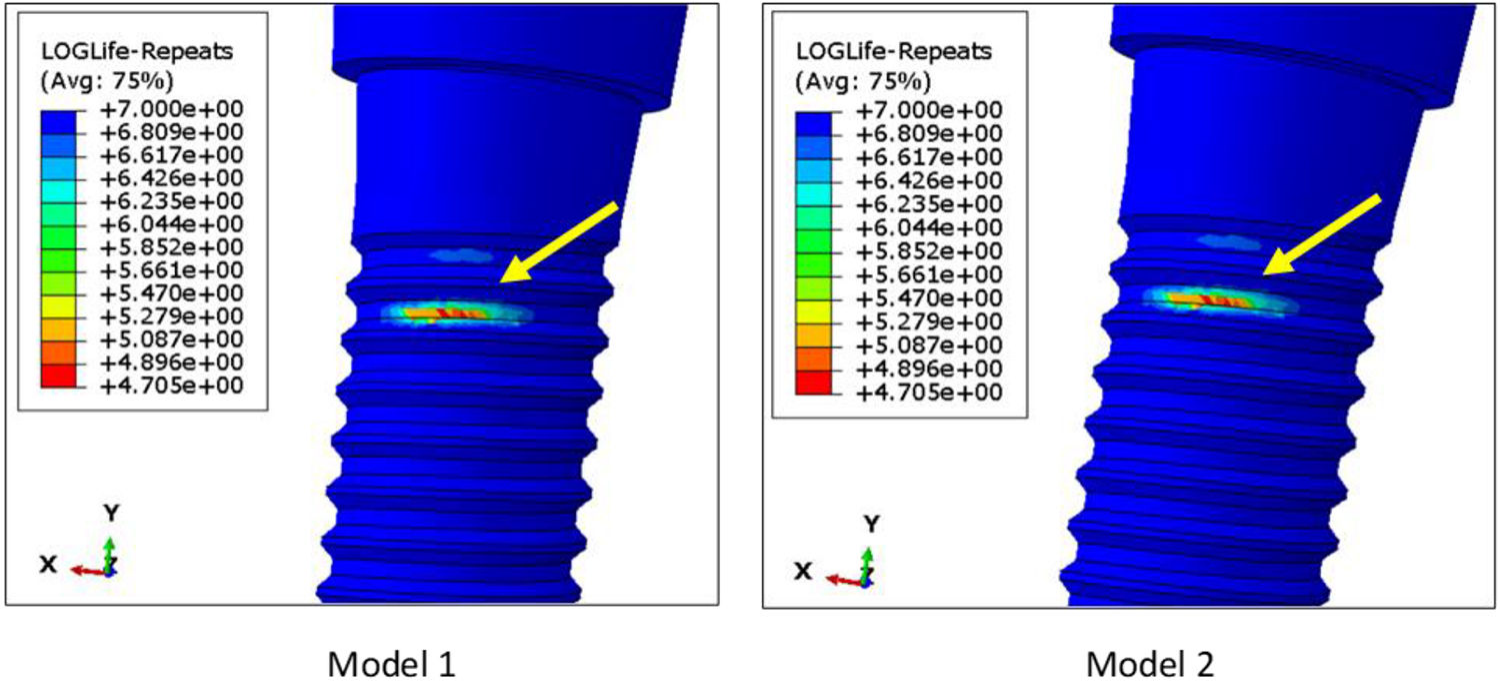
Location of fatigue failure in Models 1 and 2, as visualized in the post processor of ABAQUS. The fatigue limits for Models 1 and 2 was 116.4 N. The failure location was observed in a localized region of the superficial threads on the implant fixture. This kind of failure is very common in metals.

Under 100 N fatigue loading, the number of cycles to failure (with a mean probability of failure of 50%) for both models were infinite, as predicted by fe-safe. This type of result is expected from a commercially available dental implant, since the ISO 14801 standard requires for a dental implant to survive at least 2 million cycles under physiological loading condition. Since it was not possible to compare the fatigue lifetime of the two models at 100 N external load, we compared their fatigue limits instead. The fatigue limit calculation was also performed in fe-safe, by estimating the maximum load value for which the lifetime of the model is infinite. The fatigue limit for both Model 1 and Model 2 was 116.4 N. Failure in both the assemblies was observed at the superficial threads of the implant fixture at a localized region, which is very common in the *in vitro* [19] and *in vivo* case [20] (Figure 7). The results indicated that the remodelling of bone around a reduced-diameter dental implant does not have a clinically significant effect on the implant fatigue limit, since a difference of 0.02 N is negligible compared to other sources of variation between clinical cases. Also, the location of fatigue failure was the same in both models.

## Discussion

The space between teeth is not large enough for a standard diameter implant in some positions (mandibular incisors and maxillary lateral incisors), so reduced-diameter implants are used at these positions. However, reduced-diameter implants suffer from a higher incidence of mechanical complications. The aim of this study was to evaluate the effect of bone remodeling around a reduced-diameter dental implant on the fatigue limit predicted using 3D finite element analysis. The present study is preliminary to a study that will screen for implant design factors that have significant effects on implant fatigue limit, and this study determined that simplifying assumptions may be made in the modeling of the simulated bone holder without affecting the results of future studies.

In the clinical scenario, after the dental implant placement, bone remodeling around the implant takes place depending on the magnitude and frequency of load it experiences. In case of cortical bone remodeling, crestal bone loss takes place. For a high-quality bone, crestal bone loss up to a depth of 1 mm could be expected within the first year of implant placement, followed by an additional 0.2 mm on an average [6]. In this study, we only evaluated the effect of cancellous bone remodeling on the lifetime of a dental implant. This is because we constructed our models based on 3 mm of crestal bone resorption as required by ISO 14801 standard. One assumption of this study was that if the bone remodeling does not significantly affect the fatigue lifetime of the implant in the 3 mm resorption case, then it is less likely to have a significant effect in the case where bone resorption is less than 3 mm.

In general, if the implant fixture and abutment are two separate components, failure usually occurs at the implant fixture-abutment connection, especially in external hex implant systems [27]. This is because when a connector screw is tightened within the abutment and implant fixture, it experiences a preload, or an axial force, on its surface. This preload is essential, since it increases the probability of the screw to undergo tension-tension cycling (stress ratio > 0) instead of tension-compression (stress ratio < 0) or tension-zero (stress ratio = 0) cycling under cyclic loading. Tension-tension cycling is often less damaging than tension-compression cycling for metals [28]. When the implant is used for a period of time, there is a chance of micromotion between the implant and abutment, that can reduce the preload on the connector screw. This leads to screw loosening, that may ultimately lead to early fatigue fracture. In the present study, we did observe higher stress concentration at the connector screw, but the location of fatigue failure was observed at the superficial threads of the implant fixture. This is likely because the fatigue limit of connector screw material is 59% higher than the fatigue limit of implant fixture material.

In this study, we constructed the models based on the ISO 14801 guidelines. Hence, for all of our models, we kept the bone crest at 3 mm below the implant nominal bone level. It was important to keep the height of the bone the same in both models, not only because that is a requirement of the ISO 14801 test, but also because it has such a strong effect that it would mask any effect of bone remodeling around the implant. Since the crestal bone loss (or in other words, cortical bone remodeling) was already assumed for all of the models, we evaluated the effect of only cancellous bone remodeling on the implant fatigue lifetime.

In the present study, we used finite element analysis to predict our desired results, since it is an easier and cost-effective method for both qualitative and quantitative analysis. However, FEA studies have several limitations. For example, in this study, the material properties were assumed to be homogeneous, isotropic, and linearly elastic, but that may not necessarily reflect the practical case. Also, a lack of corrosion was assumed in our models. In the present study, the magnitude and frequency of loading was consistent throughout the analysis, but that does not reflect the clinical case, since the patient may have different bite forces and frequency of chewing when the implant is under use. Hence, the results of this study are valid for an idealistic scenario where all are assumptions are applicable. However, *in vitro* testing is conducted according to most of these assumptions, so the results of this study are significant in that they greatly simplify the computer modeling methods necessary to predict *in vitro* test results and allow more rapid exploration of a variety of implant designs.

## Conclusion

In this study, we investigated whether the remodeling of bone around a reduced-diameter dental implant affects the implant fatigue limit. Within the limitations of this study, we concluded that the remodeling of simulated bone with time around this reduced-diameter dental implant did not have a clinically significant effect on the implant fatigue limit.

## Figures and Tables

**Table 1:** Material properties assigned to the different assembly components in Model 1. The same material properties were assigned for the components in Model 2, except for the cancellous bone, where sections 1 and 2 were assigned Young’s moduli of 20 GPa and 14 GPa, respectively.

Component	Material	Young’s modulus (GPa)	Poisson’s ratio
Implant fixture	Grade 4 CPTi^1^	103	0.34
Abutment	Ti-6Al-4V^2^	110	0.31
Connector screw	316L SS^3^	180	0.30
Loading cap	316L SS^3^	180	0.30
Cancellous bone	Cancellous bone^4^	14	0.30
Cortical bone	Cortical bone^5^	20	0.30

1 [23]

2 [24]

3 [25]

4 [21]

5 [22]

**Table 2:** The number of elements for each component obtained after convergence tests.

Component	Number of elements
Implant	489,574
Abutment	421,408
Connector screw	216,077
Loading cap	1,339
Cortical bone	27,998
Cancellous bone	555,480
